# Supplementary material for: Causal Relationship between Aging and Anorexia Nervosa: A White-Matter-Microstructure-Mediated Mendelian Randomization Analysis
Source: Biomedicines. 2024 Aug 16;12(8):1874. doi: 10.3390/biomedicines12081874 (PMC11351342; doi:10.3390/biomedicines12081874)
Supplement: Supplementary file 1 [file biomedicines-12-01874-s001.zip › Supplementary Figure/Figure S8/Leaveoneout plot of MR analysis for TL to ubm-b-1505.pdf]

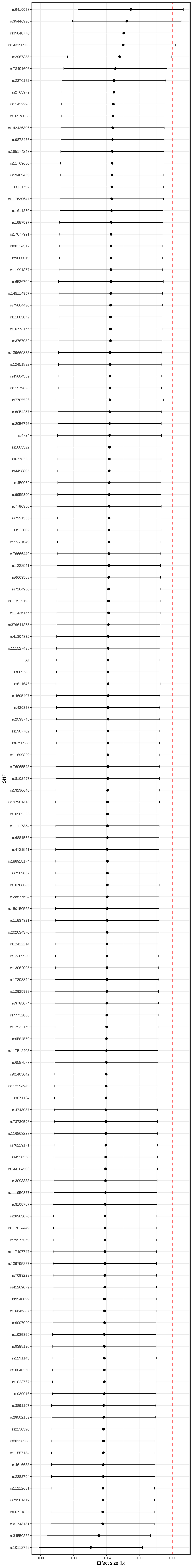
SNP
rs9419958
rs35446936
rs35640778
rs143190905
rs2967355
rs78491606
rs2276182
rs2763979
rs11412296
rs16978028
rs142426306
rs9878436
rs185174247
rs11769630
rs59409453
rs131797
rs117630647
rs1611236
rs1957937
rs17677991
rs80324517
rs9600019
rs11991877
rs6536702
rs145114957
rs75664430
rs11085072
rs10773176
rs3767952
rs139669835
rs12451892
rs45604339
rs11579626
rs7705526
rs6054257
rs2056726
rs4724
rs1003322
rs6776756
rs4498805
rs450962
rs9955360
rs7790856
rs7221585
rs932002
rs77231040
rs76666449
rs1332941
rs6669563
rs7164950
rs113525195
rs11426156
rs376641875
rs41304832
rs111527438
A8
rs869785
rs611646
rs4695407
rs429358
rs2538745
rs1907702
rs6790988
rs11699829
rs76065543
rs8102497
rs13230646
rs137901416
rs10905255
rs11117354
rs6881568
rs4731541
rs188918174
rs7209057
rs10768683
rs28577594
rs150150565
rs11584821
rs202034370
rs12412214
rs12369950
rs13062095
rs17803849
rs12925933
rs3785074
rs77732866
rs12932179
rs6584579
rs117512405
rs6587577
rs61405042
rs112394943
rs871134
rs4743037
rs73730598
rs116863223
rs76219171
rs4530278
rs144204502
rs3093888
rs111950327
rs8105767
rs28363070
rs117034449
rs79977579
rs117407747
rs139795227
rs7099229
rs41269079
rs9940099
rs10845387
rs6007020
rs1985369
rs9398196
rs1291143
rs10840270
rs1023767
rs939916
rs3891167
rs28502153
rs2230590
rs80116508
rs11557154
rs4616688
rs2282764
rs11212631
rs73581419
rs66731853
rs61748181
rs34550383
rs10112752
-0.08
-0.06
-0.04
-0.02
0.00
Effect size (b)
